# Feasibility of antiretroviral therapy initiation under the treat‐all policy under routine conditions: a prospective cohort study from Eswatini

**DOI:** 10.1002/jia2.25401

**Published:** 2019-10-24

**Authors:** Bernhard Kerschberger, Kiran Jobanputra, Michael Schomaker, Serge M Kabore, Roger Teck, Edwin Mabhena, Nomthandazo Lukhele, Barbara Rusch, Andrew Boulle, Iza Ciglenecki

**Affiliations:** ^1^ Médecins Sans Frontières (Operational Centre Geneva) Mbabane Eswatini; ^2^ Centre for Infectious Disease Epidemiology and Research School of Public Health and Family Medicine University of Cape Town Cape Town South Africa; ^3^ The Manson Unit Médecins Sans Frontières London United Kingdom; ^4^ Institute of Public Health, Medical Decision Making and HealthTechnology Assessment Medical Informatics and Technology UMIT – University for Health Sciences Hall in Tirol Austria; ^5^ Swaziland National AIDS Programme (SNAP) Ministry of Health Mbabane Eswatini; ^6^ Médecins Sans Frontières (Operational Centre Geneva) Geneva Switzerland

**Keywords:** treat all, ART initiation, linkage, Eswatini, universal ART, treatment cascade

## Abstract

**Introduction:**

The World Health Organization recommends the Treat‐All policy of immediate antiretroviral therapy (ART) initiation, but questions persist about its feasibility in resource‐poor settings. We assessed the feasibility of Treat‐All compared with standard of care (SOC) under routine conditions.

**Methods:**

This prospective cohort study from southern Eswatini followed adults from HIV care enrolment to ART initiation. Between October 2014 and March 2016, Treat‐All was offered in one health zone and SOC according to the CD4 350 and 500 cells/mm^3^ treatment eligibility thresholds in the neighbouring health zone, each of which comprised one secondary and eight primary care facilities. We used Kaplan–Meier estimates, multivariate flexible parametric survival models and standardized survival curves to compare ART initiation between the two interventions.

**Results:**

Of the 1726 (57.3%) patients enrolled under Treat‐All and 1287 (42.7%) under SOC, cumulative three‐month ART initiation was higher under Treat‐All (91%) than SOC (74%; *p *<* *0.001) with a median time to ART of 1 (IQR 0 to 14) and 10 (IQR 2 to 117) days respectively. Under Treat‐All, ART initiation was higher in pregnant women (vs. non‐pregnant women: adjusted hazard ratio (aHR) 1.96, 95% confidence interval (CI) 1.70 to 2.26), those with secondary education (vs. no formal education: aHR 1.48, 95% CI 1.12 to 1.95), and patients with an HIV‐positive diagnosis before care enrolment (aHR 1.22, 95% CI 1.10 to 1.36). ART initiation was lower in patients attending secondary care facilities (aHR 0.64, 95% CI 0.58 to 0.72) and for CD4 351 to 500 when compared with CD4 201 to 350 cells/mm^3^ (aHR 0.84, 95% CI 0.72 to 1.00). ART initiation varied over time for TB cases, with lower hazard during the first two weeks after HIV care enrolment and higher hazards thereafter. Of patients with advanced HIV disease (n = 1085; 36.0%), crude 3‐month ART initiation was similar in both interventions (91% to 92%) although Treat‐All initiated patients more quickly during the first month after HIV care enrolment.

**Conclusions:**

ART initiation was high under Treat‐All and without evidence of de‐prioritization of patients with advanced HIV disease. Additional studies are needed to understand the long‐term impact of Treat‐All on patient outcomes.

## 1 Introduction

The therapeutic effect of antiretroviral therapy (ART) and its preventive benefits of reducing HIV transmission are well established 1, 2, 3. On the basis of this evidence, the World Health Organization (WHO) established the Treat‐All policy which recommends ART initiation irrespective of CD4 cell criteria and emphasizes the prioritization of patients with advanced HIV disease 4, 5. Following these recommendations, an additional 4.1 million people living with HIV (PLHIV) required ART in Eastern and Southern Africa in 2016 6.

After HIV diagnosis, enrolment into HIV care, completion of pretreatment steps and ART initiation are important milestones in the care cascade 7, 8. Before Treat‐All, it was appreciated that loss to care of eligible patients between HIV diagnosis and ART initiation was high 9, and predictors of pretreatment losses include patient‐related, health system and clinical factors 10, 11. Community‐based linkage to care interventions, health service interventions, patient support packages and strengthened monitoring systems increased linkages and ART initiation 9, 10, 12, 13, 14, 15. While these approaches will remain important, the operational simplification of ART initiation under Treat‐All is expected to reduce pretreatment losses through shortening the pretreatment period 4, 8, 16.

These interventions are implemented in different combinations and with varying quality across HIV programmes 17, 18, and operationalization of Treat‐All may be challenging in weak health systems 19. Despite adoption of the Treat‐All policy by many countries 20, questions remain about the feasibility of ongoing ART expansion and provision of quality care in resource‐poor health settings 19, 21, with many operational challenges anticipated 22, 23.

In 2012, the Ministry of Health of Eswatini (former Swaziland) and collaborating non‐governmental organizations agreed on a national framework for the phase‐in of Treat‐All, with one such project supported by Médecins Sans Frontières (MSF). We assessed the programmatic feasibility of prompt ART initiation in all HIV‐infected patients in this public‐sector HIV programme, implemented in a single health zone while the neighbouring health zone continued with CD4 count‐based initiation criteria.

## 2 Methods

This prospective implementation study of Treat‐All was conducted in the predominantly rural Shiselweni region in southern Eswatini. HIV‐positive adults aged ≥ 16 years, who were enrolled into facility‐based HIV care from 20 October 2014 to 31 March 2016, were offered ART initiation irrespective of immunological criteria in the Treat‐All health zone. The neighbouring health zone applied national standard of care (SOC), recommending ART initiation at the CD4 cell count threshold criteria of ≤ 350 cells/mm^3^ until 31 October 2015 and ≤ 500 cells/mm^3^ thereafter. Pregnant and lactating women were eligible for prompt ART initiation in both health zones.

### 2.1 Setting

The Shiselweni region had a population of approximately 210,000 people 24, 31% of adults aged 18 to 49 years were infected with HIV 25, 26 and approximately 18,000 patients received ART in 2013 (*programme data*). The region comprised three neighbouring comparable health zones, each of which had one centralized secondary care outpatient department and eight HIV‐TB service integrated nurse‐led primary health clinics.

Although HIV testing was mainly conducted at facilities, mobile and home‐based community HIV testing was also performed routinely by MSF testing teams 27. Linkage to care interventions included short messaging appointment reminders, up to three tracing phone calls and the possibility of a home visit for patients missing their appointment. Enrolment in HIV care occurred at facility level, with the opening of a patient file and registration in the pre‐ART register. Pretreatment preparation included patient education and counselling, physical examination and screening for TB infection. Point‐of‐care CD4, haemoglobin and biochemistry testing was available at most primary care facilities. Under Treat‐All, informed written consent for prompt ART was obtained on the day of treatment initiation for patients ineligible for ART according to national standard of care (CD4 > 350 cells/mm^3^ in the absence of WHO III/IV conditions). Treatment eligible patients declining ART or patients ineligible for treatment according to national eligibility criteria were not required to sign a consent form and were retained in analysis. These patients received routine pre‐ART care and were offered ART at each clinic visit or when treatment eligibility criteria were met under SOC based on six‐monthly CD4 count testing. Same‐day ART initiation was recommended for pregnant women in both health zones. Lacking standardized operating procedures for same‐day ART initiation for non‐pregnant adults, it was undertaken at the clinician's discretion based on patient's readiness. Telephonic follow‐up was recommended for patients who missed scheduled clinic appointments.

MSF supported both health zones similarly with respect to community‐based HIV testing, linkage and facility‐based HIV‐TB care historically and during the study period 27, 28, 29. Because of the new concept of prompt ART initiation, sensitization events for communities and health workers started three months before the rollout of Treat‐All. Information and contextualized messages about Treat‐All were integrated into community‐based HIV testing activities and morning clinic talks in outpatient services. Temporarily, MSF provided first line ART drug stocks for patients with CD4 > 350 cells/mm^3^. No other additional resources were added under Treat‐All (e.g. human resources, logistics support).

### 2.2 Definitions and outcomes

Definitions of baseline covariates are provided in Table 1. Advanced HIV disease was defined as a patient presenting with CD4 cell counts <200 cells/mm^3^ and/or WHO clinical stage III/IV. The main outcome was time from facility‐based HIV care enrolment to ART initiation. Patients were right censored at the earliest of the date of the last clinic visit before transfer out of the health zone, death or database closure (31 March 2017).

**Table 1:** Baseline characteristics of patients enrolled into facility‐based HIV care for the entire treatment cohort and for patients with advanced HIV disease

(% missing values)	Entire cohort					Advanced HIV disease^a^				
	Treat‐All (n, %)		SOC (n, %)		*p* value	Treat‐All (n, %)		SOC (n, %)		*p* value
Health zone; (0)	1726		1287			631		454		
Implementation period; (0)										
Period‐1	1320	(76.5)	945	(73.4)	0.055	473	(75.0)	340	(74.9)	0.979
Period‐2	406	(23.5)	342	(26.6)		158	(25.0)	114	(25.1)	
Facility; (0)										
PHC	1068	(61.9)	801	(62.2)	0.840	393	(62.3)	243	(53.5)	0.004
SHC^b^	658	(38.1)	486	(37.8)		238	(37.7)	211	(46.5)	
Gender and pregnancy status; (1.4%)										
Non‐pregnant women	807	(47.4)	598	(47.2)	<0.001	288	(46.0)	193	(42.7)	0.006
Men	484	(28.4)	437	(34.5)		266	(42.5)	228	(50.4)	
Pregnant women	413	(24.2)	233	(18.4)		72	(11.5)	31	(6.9)	
Age at HIV care enrolment, years; (0)										
16 to 24	411	(23.8)	328	(25.5)	0.560	75	(11.9)	42	(9.3)	0.379
25 to 49	1191	(69.0)	866	(67.3)		492	(78.0)	363	(80.0)	
≥50	124	(7.2)	93	(7.2)		64	(10.1)	49	(10.8)	
Marital status; (2.7)										
Married	533	(32.1)	557	(43.8)	<0.001	233	(38.3)	222	(49.2)	<0.001
Not married	1129	(67.9)	714	(56.2)		376	(61.7)	229	(50.8)	
Education; (16.6)										
None	68	(4.9)	97	(8.6)	<0.001	33	(6.4)	42	(10.6)	0.098
Primary	336	(24.2)	359	(31.9)		140	(27.3)	114	(28.9)	
Secondary	958	(69.0)	650	(57.8)		331	(64.5)	234	(59.2)	
Tertiary	26	(1.9)	18	(1.6)		9	(1.8)	5	(1.3)	
HIV diagnosis; (1.0)										
Same day as care enrolment	881	(51.7)	756	(59.1)	<0.001	299	(47.8)	242	(53.7)	0.060
Before care enrolment	823	(48.3)	524	(40.9)		326	(52.2)	209	(46.3)	
CD4 count, cells/mm^3^; (5.3)										
0 to 100	284	(17.6)	190	(15.3)	0.342	284	(46.1)	190	(41.9)	0.279
101 to 200	272	(16.9)	204	(16.5)		270	(43.8)	204	(44.9)	
201 to 350	386	(23.9)	330	(26.7)		33	(5.4)	36	(7.9)	
351 to 500	340	(21.1)	253	(20.4)		14	(2.3)	15	(3.3)	
≥501	332	(20.6)	261	(21.1)		15	(2.4)	9	(2.0)	
WHO clinical stage; (1.8)										
I/II	1448	(86.0)	1075	(84.2)	0.194	392	(62.5)	249	(55.3)	0.029
III	213	(12.7)	175	(13.7)		213	(34.0)	175	(38.9)	
IV	22	(1.3)	26	(2.0)		22	(3.5)	26	(5.8)	
BMI, kg/m^2^; (8.5)										
≤18.4	97	(6.3)	76	(6.1)	0.313	70	(12.3)	57	(13.2)	0.672
18.5 to <25	793	(51.7)	675	(54.6)		345	(60.5)	250	(57.7)	
≥25	644	(42.0)	486	(39.3)		155	(27.2)	126	(29.1)	
Laboratory result; (16.7)										
Normal	1061	(72.5)	775	(74.0)	0.426	339	(63.5)	257	(64.7)	0.694
Abnormal	402	(27.5)	273	(26.0)		195	(36.5)	140	(35.3)	
Tuberculosis; (1.6)										
No	1612	(94.9)	1202	(94.9)	0.991	553	(88.2)	396	(88.2)	0.999
Yes	87	(5.1)	65	(5.1)		74	(11.8)	53	(11.8)	
Phone availability; (4.8)										
No	167	(10.1)	94	(7.7)	0.027	69	(11.3)	27	(6.2)	0.005
Yes	1482	(89.9)	1124	(92.3)		541	(88.7)	410	(93.8)	

Main definitions of baseline variables: A TB case was defined as a patient receiving TB treatment at the time of HIV care enrolment. Calendar time was divided into time period‐1 (20 October 2014 to 31 October 2015) and time period‐2 (01 November 2015 to 31 March 2016), corresponding to the WHO 2010 and WHO 2013 guidelines implementation periods under SOC. An abnormal baseline laboratory test result was defined as any of haemoglobin <10 g/dL, creatinine >121 μmol/L or aspartate aminotransferase (AST) >42 units/L. Same‐day HIV diagnosis was defined as an HIV‐positive diagnosis on the day of facility‐based HIV care enrolment. Patients with recorded phone numbers were considered to have access to a phone. BMI, body mass index; PHC, primary healthcare level; SHC, secondary healthcare level; SOC, standard of care.

^a^Advanced HIV disease was defined as patients presenting with CD4 <200 cells/mm3 and/or WHO III/IV staging; ^b^secondary healthcare level comprised ART outpatient departments in one health centre (with inpatient capacity) in Treat‐All and one hospital in SOC.

**Figure 1:**
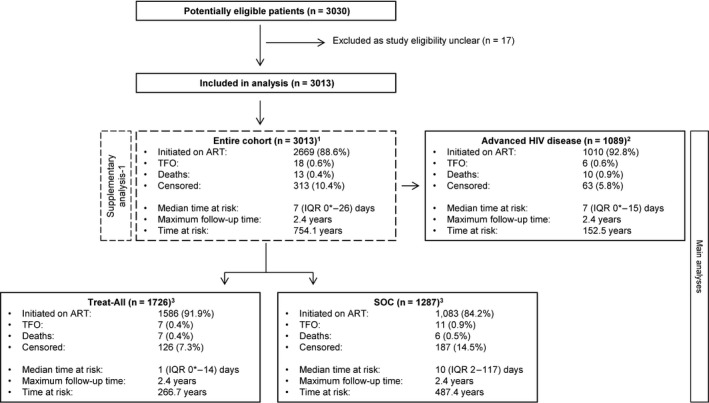
Study flow chart of main analyses. Total follow‐up time was 2.4 years in all analyses. *Zero days indicates same‐day ART initiation. ^1^The analysis directly compared Treat‐All with SOC irrespective of CD4 and WHO clinical staging criteria. ^2^The analysis directly compared Treat‐All with SOC restricted to patients with advanced HIV disease (CD<200 cells/mm^3^ and/or WHO III/IV). ^3^The Treat‐All and SOC interventions were analysed separately. ART, antiretroviral therapy; n, number; IQR, interquartile range; SOC, standard of care; TFO, transferred out.

Several analyses were performed (Figure 1). First, we compared associations with ART initiation in each of the Treat‐All and SOC zones. The analyses were done separately due to the different guidelines in operation in each, which would have made interpretation of associations and interactions with the model of care more difficult to interpret. Second, we evaluated the effect of programmatic approach (Treat‐all vs. SOC) on the outcome in both zones combined for patients presenting with advanced HIV disease. Third, in supplementary analysis‐1, a multivariate comparison of ART initiation between Treat‐All and SOC was performed for the entire cohort of patients to estimate the overall association of programmatic approach with the outcome. In supplementary analysis‐2, HIV‐positive patients diagnosed through community‐based HIV testing under Treat‐All and SOC were followed for six months to compare facility‐based care enrolment and ART initiation.

All data were collected prospectively by trained data clerks from individual patient records and facility‐based pre‐ART and ART registers. Data were entered into EpiData software and analyses were performed with Stata version 14.1 (College Station, Texas).

### 2.3 Statistics

Baseline characteristics were described using frequency statistics, proportions and medians, and compared with Wilcoxon rank‐sum and Pearson's chi‐square tests. Kaplan–Meier estimates were used to estimate and plot the crude cumulative hazard of ART initiation.

We used multiple imputation by chained equations to address missing values and performed imputation diagnostics thereafter (Table S1, Figure S1). The proportional‐hazards assumption was assessed globally and for individual variables based on Schoenfeld residuals tests in the first imputed dataset. Using 20 imputed datasets, *a priori* determined variables identified through directed acyclic graphs were included in flexible parametric survival models (Royston–Parmar models) 30. The number and locations of internal knots for the restricted cubic spline function were based on clinical, the Akaike's and Schwarz's Bayesian information, and graphical criteria. Standardized failure curves and contrasts were plotted after fitting Royston–Parmar models 31, 32.

### 2.4 Ethics

The findings reported here are part of a larger study assessing the feasibility of Treat‐All under routine conditions. The study and analyses were approved by the Research Ethics Committees of MSF, the Scientific and Ethics Committee of Eswatini and the University of Cape Town, South Africa.

## 3 Results

### 3.1 Baseline characteristics

Seventeen patients were excluded from analysis as study eligibility criteria were unclear (Figure 1). Of the remaining 3013 patients enrolled into facility‐based HIV care, 1726 (57.3%) were in Treat‐All and 1287 (42.7%) in SOC (Table 1). Excluding missing values of covariates (Table S1), 1869 (62.0%) patients presented at primary care facilities, the median age and CD4 cell count were 30 (interquartile range (IQR) 25 to 37) years and 297.5 (IQR 153 to 467) cells/mm^3^, 921 (31.0%) were men, 1637 (54.9%) were diagnosed on the day of HIV care enrolment, 436 (14.7%) had WHO III/IV clinical staging, and 152 (5.1%) had TB.

Patients under Treat‐All were more likely to be pregnant women (24.2% vs. 18.4%, *p *<* *0.001), diagnosed HIV positive before care enrolment (48.3% vs. 40.9%, *p *<* *0.001), without access to a phone (10.1% vs. 7.7%, *p *=* *0.027), unmarried (67.9% vs. 56.2%, *p *<* *0.001), and had higher education (e.g. secondary education 69.0% vs. 57.8%, *p *<* *0.001).

### 3.2 Crude ART initiation

Crude outcome data and survival‐time data of the main analyses are presented in Figure 1.

**Figure 2:**
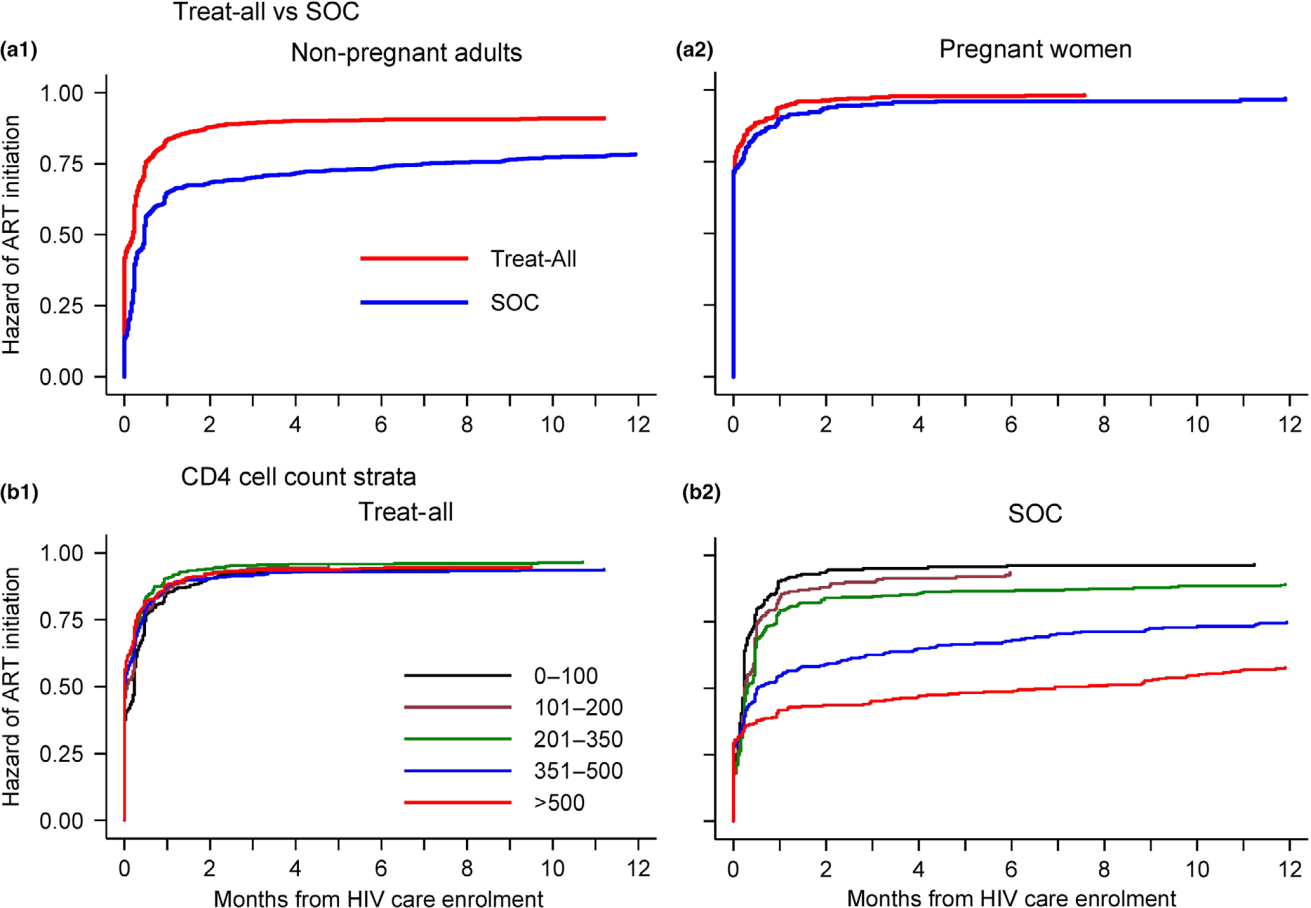
Crude Kaplan–Meier estimates of time to ART initiation since HIV care enrolment for (a) Treat‐All and SOC by pregnancy status and (b) CD4 cell strata by intervention. SOC, standard of care. As most ART initiations occur within 12 months, the plotted curves are graphed only for this period.

Most ART initiations occurred within two months after HIV care enrolment (Figure 2). Median time to ART was 1 (IQR 0 to 14) day under Treat‐All and 10 (IQR 2 to 117) days under SOC. Crude cumulative three‐month ART initiation was 91% and 74% respectively (*p *<* *0.001). Three‐month ART initiation was highest among pregnant women and similar between Treat‐All (97%) and SOC (95%) (*p *=* *0.220) (Figure 2, a1‐a2). Crude ART initiations were similar across CD4 cell strata under Treat‐All (*p *=* *0.075), but lower for CD4 351 to 500 and CD4 ≥ 501 cells/mm^3^ under SOC (*p *<* *0.001) (Figure 2b1‐b2).

ART initiation on the day of HIV care enrolment (same‐day ART) was similarly high for pregnant women under Treat‐All (71%) and SOC (72%) (*p* = 0.220). It was higher for non‐pregnant adults under Treat‐All (42%) than SOC (14%) (*p* < 0.001) (Figure 2a1‐a2).

### 3.3 Predictors of ART initiation

#### 3.3.1 Treat‐all

**Figure 3:**
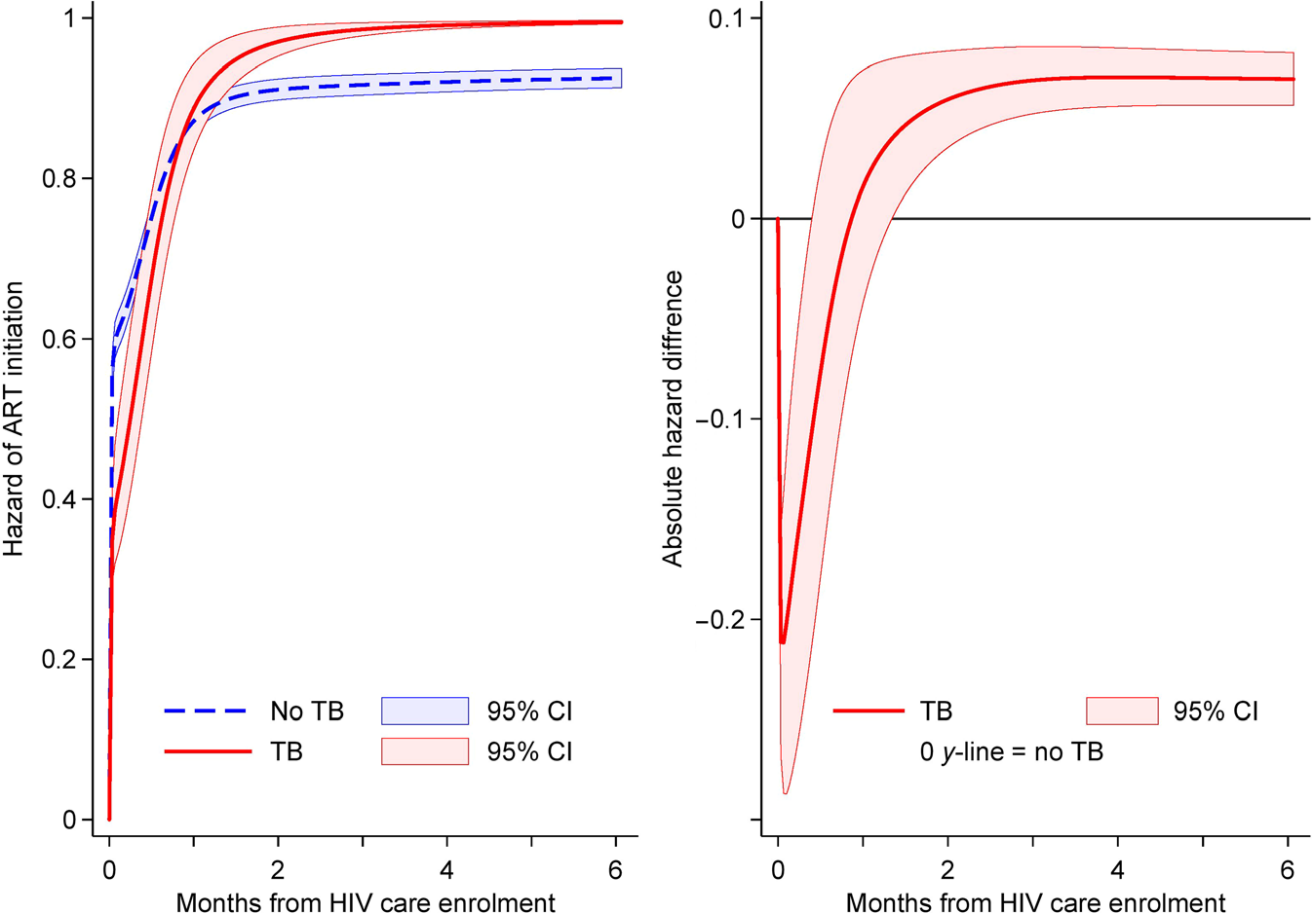
Cumulative hazard of ART initiation and absolute difference in hazard of ART initiation by TB status under Treat‐All. After fitting the covariate‐adjusted flexible parametric survival model for Treat‐All (see Table 2), we estimated the standardized failure curves for patients with and without TB under Treat‐All and the contrasts using the post estimation Stata command *stpm2_standsurv* 31, 32. CI, confidence interval.

Among patients enrolling under Treat‐All, the hazard of ART initiation was higher for time period‐2 (adjusted hazard ratio (aHR) 1.22, 95% confidence interval (CI) 1.08 to 1.38), for pregnant women (aHR 1.96, 95% CI 1.70 to 2.26) versus non‐pregnant women, for patients with secondary education (aHR 1.48, 95% CI 1.12 to 1.95) versus no formal education, and for HIV‐positive diagnoses before HIV care enrolment (aHR 1.22, 95% CI 1.10 to 1.36) (Table 2). ART initiation was lower for the secondary care facility (aHR 0.64, 95% CI 0.58 to 0.72) and tended to be lower for patients with CD4 351 to 500 cells/mm^3^ (aHR 0.84, 95% CI 0.72 to 1.00) versus CD4 201 to 350 cells/mm^3^. Although TB co‐infection had a similar overall hazard of ART initiation when compared with no TB (Table 2), the effect varied over time with lower hazard during the first two weeks after HIV care enrolment and higher hazards thereafter (Figure 3).

**Table 2:** Predictors of ART initiation for all patients enrolled into HIV care under Treat‐All and standard of care (SOC)

	Treat‐All (n = 1726)				SOC (n = 1287)			
	HR	95% CI	aHR	95% CI	HR	95% CI	aHR	95% CI
Implementation period								
Period‐1	1		1		1		1	
Period‐2	1.25	(1.11 to 1.40)	1.22	(1.08 to 1.38)	1.28	(1.12 to 1.47)	1.28	(1.11 to 1.47)
Facility								
PHC	1		1		1		1	
SHC^a^	0.66	(0.60 to 0.73)	0.64	(0.58 to 0.72)	1.63	(1.44 to 1.84)	1.39	(1.21 to 1.59)
Gender and pregnancy status								
Non‐pregnant women	1		1		1		1	
Men	0.92	(0.82 to 1.04)	0.93	(0.81 to 1.07)	1.13	(0.99 to 1.29)	1.04	(0.90 to 1.22)
Pregnant women	1.88	(1.65 to 2.13)	1.96	(1.70 to 2.26)	3.38	(2.86 to 3.99)	3.50	(2.87 to 4.25)
Age at HIV care enrolment, years								
16 to 24	1		1		1		1	
25 to 49	0.90	(0.80 to 1.01)	1.03	(0.91 to 1.18)	1.04	(0.90 to 1.19)	0.97	(0.83 to 1.14)
≥50	0.78	(0.63 to 0.96)	1.05	(0.82 to 1.34)	0.93	(0.72 to 1.20)	0.99	(0.75 to 1.30)
Marital status								
Married	1		1		1		1	
Not married	1.01	(0.91 to 1.13)	1.01	(0.89 to 1.14)	1.05	(0.92 to 1.18)	1.07	(0.93 to 1.23)
Education								
None	1		1		1		1	
Primary	1.35	(1.01 to 1.79)	1.32	(0.98 to 1.77)	0.99	(0.78 to 1.26)	0.93	(0.72 to 1.20)
Secondary	1.57	(1.20 to 2.04)	1.48	(1.12 to 1.95)	1.05	(0.84 to 1.32)	0.85	(0.67 to 1.09)
Tertiary	1.56	(0.97 to 2.50)	1.50	(0.89 to 2.54)	1.46	(0.84 to 2.52)	1.01	(0.58 to 1.74)
HIV diagnosis								
Same day as care enrolment	1		1		1		1	
Before care enrolment	1.05	(0.95 to 1.16)	1.22	(1.10 to 1.36)	1.24	(1.09 to 1.39)	1.28	(1.12 to 1.45)
CD4 count, cells/mm^3^								
0 to 100	0.78	(0.66 to 0.93)	0.90	(0.74 to 1.10)	1.41	(1.17 to 1.71)	1.42	(1.14 to 1.77)
101 to 200	0.87	(0.74 to 1.03)	0.85	(0.72 to 1.01)	1.17	(0.97 to 1.41)	1.12	(0.92 to 1.36)
201 to 350	1		1		1		1	
351 to 500	0.86	(0.74 to 1.02)	0.84	(0.72 to 1.00)	0.66	(0.55 to 0.80)	0.67	(0.56 to 0.81)
≥501	0.91	(0.76 to 1.08)	0.86	(0.71 to 1.03)	0.49	(0.41 to 0.59)	0.48	(0.40 to 0.59)
WHO clinical stage								
I/II	1		1		1		1	
III	0.76	(0.65 to 0.90)	0.87	(0.72 to 1.07)	1.41	(1.19 to 1.68)	1.04	(0.84 to 1.28)
IV	0.61	(0.39 to 0.97)	0.77	(0.48 to 1.24)	1.48	(0.99 to 2.22)	0.99	(0.63 to 1.55)
BMI, kg/m^2^								
≤18.4	1		1		1		1	
18.5 to <25	1.06	(0.84 to 1.34)	0.88	(0.68 to 1.14)	0.73	(0.57 to 0.94)	0.85	(0.64 to 1.13)
≥25	1.25	(0.98 to 1.61)	0.86	(0.64 to 1.15)	0.81	(0.62 to 1.05)	0.89	(0.67 to 1.20)
Laboratory result								
Normal	1		1		1		1	
Abnormal	1.03	(0.86 to 1.24)	1.13	(0.93 to 1.37)	1.28	(1.06 to 1.55)	1.13	(0.91 to 1.40)
Tuberculosis								
No	1		1		1		1	
Yes	0.79	(0.63 to 0.98)	0.86	(0.56 to 1.34)	1.25	(0.97 to 1.61)	1.06	(0.74 to 1.51)
Phone availability								
No	1		1		1		1	
Yes	1.23	(1.03 to 1.47)	1.07	(0.89 to 1.29)	1.39	(1.07 to 1.61)	1.20	(0.92 to 1.58)

The flexible parametric models (Royston–Parmar models) for Treat‐All and SOC had each five internal knots in addition to one internal knot for the time‐varying covariate tuberculosis. All variables tested in univariate analysis were included in multivariate analysis. aHR, adjusted hazard ratio; BMI, body mass index; HR, hazard ratio; PHC, primary healthcare level; SHC, secondary healthcare level; SOC, standard of care.

^a^Secondary healthcare level comprised ART outpatient departments in one health centre with inpatient capacity in Treat‐All and one hospital in SOC.

**Figure 4:**
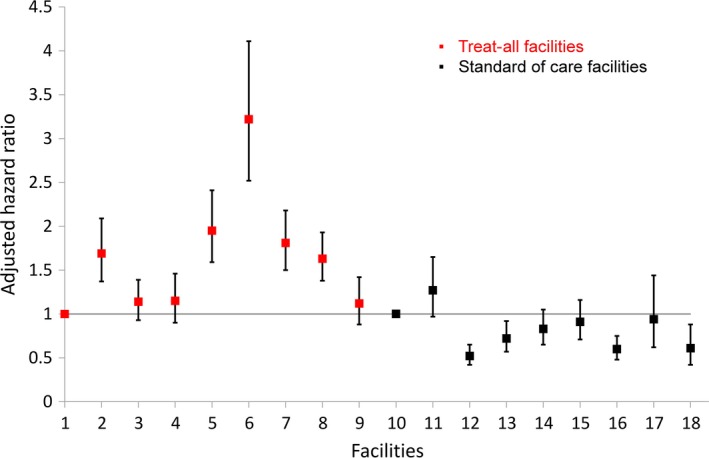
Variations in adjusted hazard ratios of ART initiation comparing primary care facilities with the secondary care facility under Treat‐All (facility 1) and under standard of care (facility 10).

Modelling with the facility as covariate (instead of healthcare level), the hazard of ART initiation in primary care clinics compared with the secondary facility was higher for five out of eight primary care clinics, with aHRs ranging from 1.63 to 3.22 (Figure 4).

#### 3.3.2 Standard of care

Among patients enrolling under SOC, the time period‐2 and timing of HIV diagnosis showed a similar increase in hazard of ART initiation to that under Treat‐All, but this was more pronounced for pregnant women (aHR 3.50, 95% CI 2.87 to 4.25) (Table 2). In contrast to Treat‐All, ART initiation was higher for the secondary care facility (aHR 1.39, 95% CI 1.21 to 1.59), while no association was detected for education level. Compared with CD4 cell count 201 to 350 cells/mm^3^ and in contrast to Treat‐All, the hazard of ART initiation was higher for low CD4 ≤ 100 (aHR 1.42, 95% CI 1.14 to 1.77), and it was lower for CD4 350 to 500 (aHR 0.67, 95% CI 0.56 to 0.81) and CD4 ≥ 500 (aHR 0.48, 95% CI 0.40 to 0.59) cells/mm^3^. The time‐varying effect of TB was less pronounced than under Treat‐All (*data not shown*).

Taking the facility covariate into account, the hazard of ART initiation was lower for four out of eight primary care clinics compared with the secondary care facility, with aHRs ranging from 0.52 to 0.74 (Figure 4).

### 3.4 Advanced HIV disease

Of 1085/3013 (36.0%) complete observations for patients with advanced HIV disease, 631 (58.2%) presented under Treat‐All and 454 (41.8%) under SOC (Table 1). The overall median CD4 cell count was 112.5 (IQR 56 to 170) cells/mm^3^, and 436 (40.5) had WHO III/IV clinical stage. Comparing the two interventions, differences in the distribution of baseline characteristics were seen for facility, gender and pregnancy status, marital status, WHO clinical staging and phone availability (Table 1).

**Figure 5:**
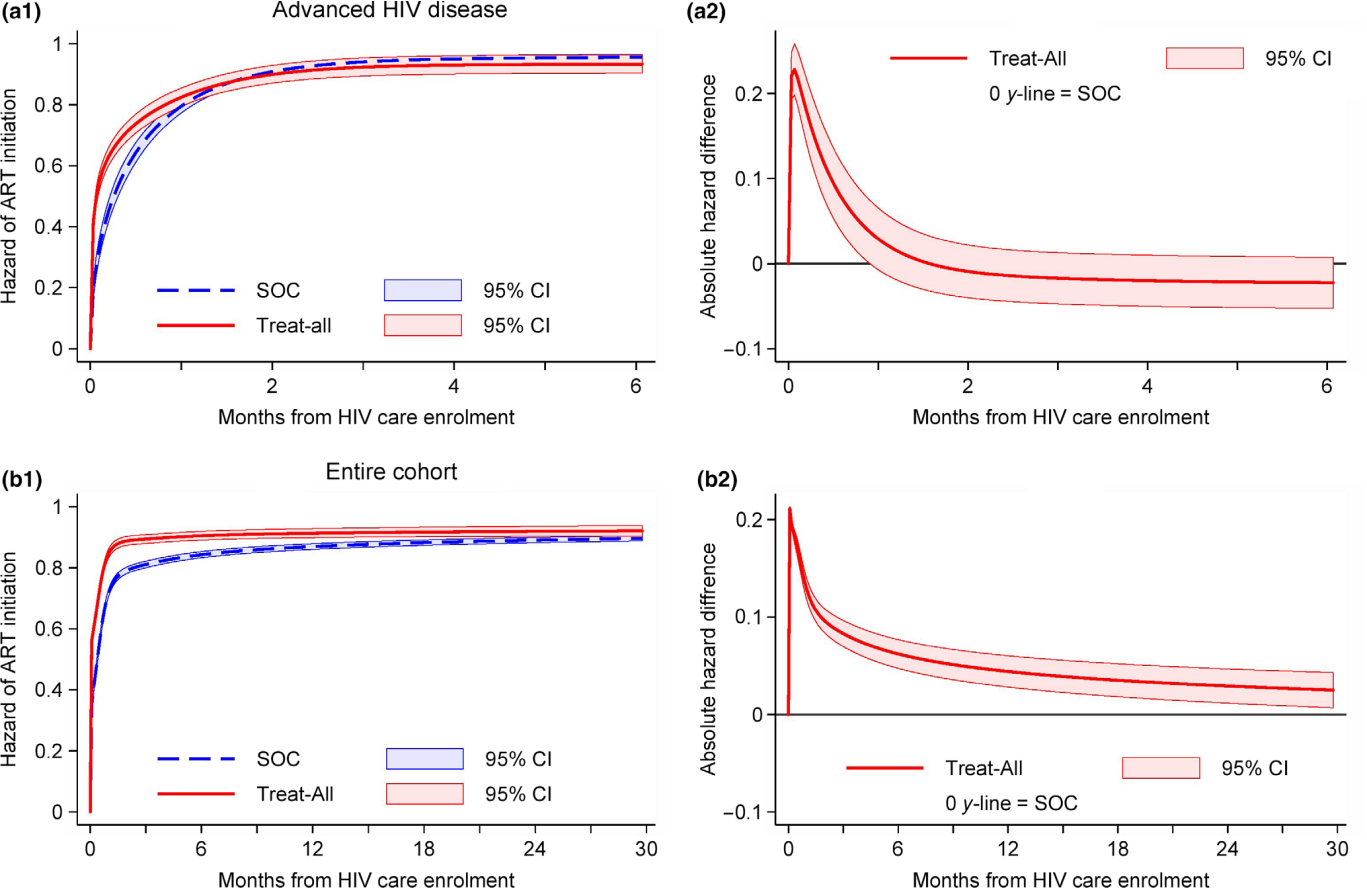
Cumulative hazard of ART initiation and absolute difference in hazard of ART initiation by intervention for patients with advanced HIV disease (a1‐2) and for the entire cohort (b1‐2). After fitting the covariate‐adjusted flexible parametric survival models for patients with advanced HIV disease (see Table 3) and for the entire cohort (see Table S2), we estimated the standardized failure curves for Treat‐All and SOC and the contrasts (Treat‐All vs. SOC) using the post estimation Stata command *stpm2_standsurv* 31, 32. CI, confidence interval; SOC, standard of care.

Crude same‐day ART initiation was higher under Treat‐All (42%) than SOC (17%) while three‐month ART initiation was similar (Treat‐All: 92%; SOC: 91%). In multivariate analysis after multiple imputations (n = 1089) (Table 3), Treat‐All had a higher hazard of ART initiation (aHR 1.75, 95% CI 1.47 to 2.08). The effect varied over time, with Treat‐All initiating patients more quickly during the first month (Figure 5a1‐2). As in previous analyses, ART initiation was higher for time period‐2, pregnant women and HIV‐positive diagnosis before HIV care enrolment while it was lower for secondary health care level. The overall hazard of ART was lower in TB cases (aHR 0.65, 95% CI 0.49 to 0.86) and the effect varied over time (*data not shown*). No associations were seen for CD4 cell count.

**Table 3:** Predictors of ART initiation for patients with advanced HIV disease

	Univariate (n = 1089)		Multivariate (n = 1089)	
	HR	95% CI	aHR	95% CI
Health zone				
SOC	1		1	
Treat‐all	1.23	(1.08 to 1.39)	1.75	(1.47 to 2.08)
Implementation period				
Period‐1	1		1	
Period‐2	1.28	(1.11 to 1.48)	1.35	(1.17 to 1.57)
Facility				
PHC	1		1	
SHC^a^	0.86	(0.76 to 0.98)	0.86	(0.75 to 0.98)
Gender and pregnancy status				
Non‐pregnant women	1		1	
Men	1.02	(0.89 to 1.16)	1.04	(0.90 to 1.21)
Pregnant women	2.02	(1.62 to 2.50)	1.90	(1.50 to 2.41)
Age at HIV care enrolment, years				
16 to 24	1		1	
25 to 49	0.96	(0.78 to 1.17)	1.02	(0.82 to 1.27)
≥50	0.89	(0.68 to 1.17)	1.01	(0.75 to 1.36)
Marital status				
Married	1		1	
Not married	0.98	(0.86 to 1.12)	0.97	(0.85 to 1.12)
Education				
None	1		1	
Primary	1.00	(0.74 to 1.36)	0.90	(0.66 to 1.22)
Secondary	1.11	(0.84 to 1.46)	0.96	(0.71 to 1.28)
Tertiary	1.27	(0.68 to 2.39)	0.93	(0.50 to 1.74)
Time of HIV diagnosis, days				
Same day	1		1	
Before	1.19	(1.05 to 1.35)	1.31	(1.15 to 1.49)
CD4 count, cells/mm^3^				
0 to 100	1.28	(0.97 to 1.69)	1.23	(0.89 to 1.70)
101 to 200	1.27	(0.96 to 1.68)	1.11	(0.79 to 1.57)
201 to 350	1		1	
351 to 500	0.94	(0.58 to 1.51)	0.91	(0.55 to 1.50)
≥501	0.95	(0.56 to 1.61)	0.88	(0.49 to 1.57)
WHO clinical stage				
I/II	1		1	
III	0.85	(0.75 to 0.97)	0.96	(0.81 to 1.14)
IV	0.76	(0.56 to 1.04)	0.83	(0.60 to 1.16)
BMI, kg/m^2^				
≤18.4	1		1	
18.5 to <25	1.03	(0.84 to 1.26)	1.01	(0.81 to 1.25)
≥25	1.14	(0.90 to 1.43)	1.03	(0.79 to 1.34)
Laboratory result				
Normal	1		1	
Abnormal	1.01	(0.86 to 1.20)	1.05	(0.88 to 1.25)
Tuberculosis				
No	1		1	
Yes	0.90	(0.74 to 1.08)	0.65	(0.49 to 0.86)
Phone availability				
No	1		1	
Yes	1.24	(0.98 to 1.56)	1.18	(0.91 to 1.53)

The flexible parametric model (Royston–Parmar models) had four internal knots, and three internal knots for the time‐varying covariates health zone and TB. All variables tested in univariate analysis were also included in multivariate analysis. aHR, adjusted hazard ratio; BMI, body mass index; HR, hazard ratio; PHC, primary healthcare level; SHC, secondary healthcare level; SOC, standard of care.

^a^Secondary healthcare level comprised ART outpatient departments in one health centre with inpatient capacity in Treat‐All and one hospital in SOC.

### 3.5 Supplementary analyses

#### 3.5.1 Supplementary analysis‐1

Considering all patients irrespective of CD4 cell and WHO clinical staging criteria, the overall hazard of ART initiation was higher under Treat‐All than SOC (aHR 1.99, 95% CI 1.81 to 2.19) (Table S2). The hazard varied over time, being highest during the first three months after HIV care enrolment (Figure 5b1‐2), steadily decreasing thereafter but remaining above SOC during the entire observation period. Associations of other covariates were similar as in previous analyses (Table S2).

#### 3.5.2 Supplementary analysis‐2

**Figure 6:**
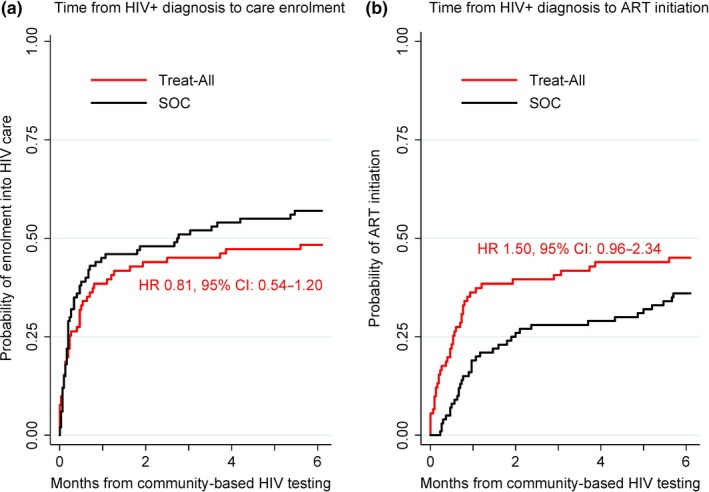
Time from community‐based HIV‐positive diagnosis under Treat‐All and standard of care (SOC) (a) to HIV care enrolment and (b) to antiretroviral therapy initiation. ART, antiretroviral theraphy; CI, confidence interval Hr, crude hazard ratio comparing outcomes under treat‐All vs SOC; SOC, standard of care.

Linkage outcomes from the time of community‐based HIV‐positive diagnosis are presented in Figure 6. Patients known to be transferred between Treat‐All and SOC were removed from the analysis (n = 10). Of the remaining 191 patients, 91 (47.6%) were diagnosed under Treat‐All and 100 (52.4%) under SOC. The median days to HIV care enrolment were similar (Treat‐All: 7, IQR 2.5 to 22; SOC: 6, IQR 3 to 21; *p *=* *0.651), but the median number of days to ART initiation was lower under Treat‐All (15, IQR 4 to 24) than SOC (29, IQR 18 to 67) (*p *=* *0.001). Six‐month HIV care enrolment was 48% under Treat‐All and 57% under SOC (*p* = 0.286), and six‐month ART initiation was 45% and 36% respectively (*p* = 0.078). The crude hazard of HIV care enrolment from the time of HIV‐positive diagnosis was similar (HR 0.81, 95% CI 0.54 to 1.20) between Treat‐All and SOC, while Treat‐All tended to have a higher hazard of ART initiation (HR 1.50, 95% CI 0.96 to 2.34).

## 4 Discussion

We assessed the feasibility of ART initiation under the Treat‐All policy in a routine public sector setting in rural Eswatini. Overall, facility‐based ART initiation was quicker and higher under Treat‐All than SOC and it did not result in de‐prioritisation of patients with advanced HIV disease. Despite suboptimal linkage, patients who were diagnosed with HIV in the community were also more likely to initiate ART under Treat‐all.

### 4.1 Findings in context

We found higher ART initiation under Treat‐All than SOC, overall and for patients presenting with CD4 ≥ 351 cells/mm^3^. SOC restricted ART eligibility for non‐pregnant adults to CD4 ≤ 350 and ≤ 500 cells/mm^3^ and/or WHO III/IV clinical staging 33. The often‐seen steep gradient in ART initiation by CD4 count, which was still present in SOC, was not seen under Treat‐All, suggesting compliance with the new policy. When the analysis, however, was restricted to Treat‐All, patients with CD4 ≥ 351 cells/mm^3^ tended to have a lower hazard of ART initiation. While a Treat‐All trial in South Africa reported similar three‐month ART uptake across CD4 strata 34, increased patient tracking was needed for patients with higher CD4 cell counts in East Africa 35. In this setting, there was reportedly less focus on patient tracking during the pretreatment period, possibly explaining this finding.

Men had the same hazard of ART initiation as non‐pregnant women in both interventions, similar to findings from an universal ART setting in South Africa 34. Notably, pregnant women were more likely to initiate ART overall and same‐day. Same‐day ART initiation was emphasized in pregnant women, while ART could be deferred for non‐pregnant adults depending on the patient's readiness 36.

Patients diagnosed on the same day as enrolment for HIV care were less likely to initiate treatment in both interventions, possibly related to internalized stigma when receiving an HIV diagnosis 37, 38.

Data suggested that patients under Treat‐All who have had secondary education may be more likely to initiate ART. This may be a chance finding given the lack of association in all other analyses and in other Treat‐All trials 34, 35.

The time period‐2 was associated with higher ART initiation in both interventions. Associations of temporal trends with patient outcomes have been reported previously from sub‐Saharan Africa 39, 40, 41, 42, 43, 44, 45, 46. First, in our setting, expanding treatment eligibility increased the pool of treatment eligible patients, likely resulting in increasing ART initiations under SOC. In addition, patients ineligible for treatment also initiated ART under SOC, although this phenomenon was not further analysed. Second, national‐level policy change on treatment eligibility allowed for increased mobilization, which may have influenced patients’ and health workers’ acceptance of early treatment under Treat‐All.

While under Treat‐All, two‐thirds of primary care clinics had a higher hazard of ART initiation (vs. secondary care facility), it was lower in one‐third of primary care clinics under SOC. Clinic, health worker and patient level factors have been reported to influence pre‐treatment losses 14, 38, 47, possibly affecting facilities under Treat‐All and SOC differently.

### 4.2 Advanced HIV disease

We showed that immunocompromised patients initiated ART more quickly under Treat‐All, possibly due to higher rates of same‐day ART initiation under Treat‐All than SOC. Same‐day ART initiation has been shown to decrease pretreatment losses 15, and rapid ART initiation is recommended by WHO 5.

ART initiation in TB cases was delayed during the first two weeks after HIV care enrolment, although overall three‐month treatment initiation was higher. First, TB needs to be ruled out before ART initiation and TB diagnosis was likely delayed. Sputum samples of presumptive TB cases were sent for Xpert MTB/RIF testing to the centralized health centres, likely increasing treatment turnaround time. Second, ART initiation in TB patients is recommended after TB treatment is started due to toxicity concerns 4, preferably within two weeks of starting TB treatment in Eswatini 36.

### 4.3 Linkages to HIV care

Suboptimal linkage is a main weakness in the HIV care cascade 9, 27, 48. Of PLHIV diagnosed at community level under Treat‐All, only 48% enrolled into care and 45% initiated ART. Comparable six‐month outcomes were reported from the TasP (47.6% linkage) 49 and PopART (42% initiating ART) 50 trials, in contrast to the SEARCH trial with 86% of patients having had at least one clinic visit within 12 months 35. Despite higher ART initiation under Treat‐All, it remained suboptimal, potentially compromising the population‐level effect of reduction in HIV incidence 51. However, a peer‐delivered linkage case management intervention in Eswatini has shown potential for high linkage and ART initiation (96%) under routine conditions in Treat‐All 52.

In this context, community‐based HIV testing played less of a role in diagnosing PLHIV (approximately 20% of all HIV‐positive diagnoses) 53. Thus, suboptimal linkages are likely to have a smaller impact on the pretreatment cascade.

### 4.4 Limitations and strengths

First, the treatment initiation rate was likely overestimated. As reliable patient‐level data on linkage to HIV care were lacking, the analysis time started from the time of facility‐based HIV care enrolment. In Eswatini, an estimated 83% to 92% of HIV‐positive diagnosed patients link from facility‐based HIV testing to care 54. Second, we did not assess ART outcomes, which was beyond the scope of this analysis. Estimating long‐term treatment outcomes is crucial to understand the feasibility of Treat‐All in the wider context. Third, the intervention areas were chosen purposely to ensure comparability in clinical practice. Both zones were located in the same geographic area, received the same support and had the same number of primary and secondary care facilities. Nevertheless, the analysis indicated differences in baseline characteristics of patients and inter‐facility variations in ART initiation, highlighting possible variations in patients' behaviour and clinical practice. Despite adjusting for it in analyses, there may be still differences with respect to unobserved variables. Lastly, using data from a routine setting may compromise data quality and completeness.

External validity and generalizability is a strength of this study, as this setting has characteristics of many resource‐limited settings in rural sub‐Saharan Africa. Treat‐All was introduced into a routine government HIV programme. The policy change towards Treat‐All built on previous operational gains. For example, more than half of all patients enrolled at decentralized primary care clinics with a high degree of HIV‐TB service integration. Only trainings and information were provided in addition to routine activities and the Ministry of Health started providing the additional drugs during the study period. Although this study does not address all aspects of feasibility (e.g. lack of economic evaluation), we believe it contributes to the ongoing discussion on programmatic feasibility of treatment expansion in resource‐poor settings.

## 5 Conclusions

Although direct comparisons of Treat‐All and SOC was limited due to possible unmeasured differences between health zones, Treat‐All appeared feasible in this public‐sector HIV programme. Overall, ART initiation was high and without de‐prioritization of patients with advanced HIV disease. Despite suboptimal linkage to care, more PLHIV initiated ART under Treat‐All than SOC.

## Competing interest

The authors declare no conflict of interest.

## Authors’ contributions

BK, KJ and RT designed the study. BK and JK established the cohort and were involved in data acquisition. BK, MS, AB and IC led the data analysis plan. BK performed the statistical analyses and wrote the first draft of the manuscript. MS and AB advised on final analyses. BK, KJ, MS, SMK, EM, NL, AB and IC interpreted the data. All authors contributed to the manuscript and approved the final version.

## Supporting information


**Table S1.** Covariates with missing values
**Table S2.** Multivariate comparison of ART initiation between Treat‐All and SOC for the entire cohort of patients enrolled into facility‐based HIV care
**Figure S1.** Trace plots of covariates with missing values.
