# Supplementary material for: Feasibility of antiretroviral therapy initiation under the treat‐all policy under routine conditions: a prospective cohort study from Eswatini
Source: J Int AIDS Soc. 2019 Oct 24;22(10):e25401. doi: 10.1002/jia2.25401 (PMC6812490; doi:10.1002/jia2.25401)
Supplement: Supplementary file 1 — Table S1. Covariates with missing values Table S2. Multivariate comparison of ART initiation between Treat‐All and SOC for the entire cohort of patients enrolled into facility‐based HIV care Figure S1. Trace plots of covariates with missing values. [file JIA2-22-e25401-s001.docx]

**Supplementary File**

Study:

Feasibility of antiretroviral therapy initiation under the Treat-All policy under routine conditions: a prospective cohort study from Eswatini

Bernhard Kerschberger^1,2^, Kiran Jobanputra^3^, Michael Schomaker^2,4^, Serge M Kabore^1^, Roger Teck^3^, Edwin Mabhena^1^, Nomthandazo Lukhele^5^, Barbara Rusch^6^, Andrew Boulle^2^, Iza Ciglenecki^6^

*1 Médecins Sans Frontières (Operational Centre Geneva), Mbabane, Eswatini*

*2 Centre for Infectious Disease Epidemiology and Research, School of Public Health and Family Medicine, University of Cape Town, Cape Town, South Africa*

*3 The Manson Unit, Médecins Sans Frontières, London, United Kingdom*

*4 Institute of Public Health, Medical Decision Making and HealthTechnology Assessment, UMIT - University for Health Sciences, Medical Informatics and Technology, Hall in Tirol, Austria*

*5 Swaziland National AIDS Programme (SNAP), Ministry of Health, Mbabane, Eswatini*

*6 Médecins Sans Frontières (Operational Centre Geneva), Geneva, Switzerland*

1. **Multiple imputation diagnostic**

As implemented in Stata version 14.1 (College Station, Texas), multiple imputation by chained equation was used to impute missing values of covariates. Table S1 shows the absolute number and proportion of missing values by covariate.

**Table S1**: Covariates with missing values.

|  | Missing values^1^ | |
| --- | --- | --- |
|  | Number | Percentage |
| CD4 count | 161 | 5.3% |
| Body mass index | 255 | 8.5% |
| WHO clinical stage | 54 | 1.8% |
| Education | 501 | 16.6% |
| Time of HIV diagnosis | 29 | 1.0% |
| Gender and pregnancy status | 41 | 1.4% |
| Marital status | 80 | 2.7% |
| Laboratory result | 502 | 16.7% |
| Phone availability | 146 | 4.8% |
| Tuberculosis | 47 | 1.6% |

*^1^The total number of observations (denominator) was 3013.*

A total of 20 imputed datasets was created. Imputation diagnostic was performed comparing the distributions of the observed, imputed and completed values. In addition, trace plots were inspected to assess whether imputation for covariates reached convergence. Imputation diagnostics were satisfied and Figure S1 presents the trace plot.

**Figure S1:** Trace plots of covariates with missing values.

1. **Additional results**

**Table S2**: Multivariate comparison of ART initiation between Treat-All and SOC for the entire cohort of patients enrolled into facility-based HIV care.

|  | **Univariate (n= 3013)** | | **Multivariate (n= 3013)** | |
| --- | --- | --- | --- | --- |
|  | **HR** | **95% CI** | **aHR** | **95% CI** |
| **Health zone** |  |  |  |  |
| SOC | 1 |  | 1 |  |
| Treat-All | 1.73 | (1.60 to 1.87) | 1.99 | (1.81 to 2.19) |
| **Implementation period** |  |  |  |  |
| Period-1 | 1 |  | 1 |  |
| Period-2 | 1.21 | (1.11 to 1.32) | 1.26 | (1.16 to 1.38) |
| **Facility** |  |  |  |  |
| PHC | 1 |  | 1 |  |
| SHC^1^ | 1.01 | (0.93 to 1.09) | 0.90 | (0.82 to 0.97) |
| **Gender and pregnancy status** |  |  |  |  |
| Non-pregnant women | 1 |  | 1 |  |
| Men | 0.99 | (0.91 to 1.09) | 0.96 | (0.87 to 1.06) |
| Pregnant women | 2.47 | (2.23 to 2.73) | 2.46 | (2.21 to 2.75) |
| **Age at HIV care enrolment, years** |  |  |  |  |
| 16 to 24 | 1 |  | 1 |  |
| 25 to 49 | 0.97 | (0.89 to 1.06) | 1.02 | (0.92 to 1.13) |
| ≥50 | 0.85 | (0.72 to 1.00) | 1.01 | (0.84 to 1.20) |
| **Marital status** |  |  |  |  |
| Married | 1 |  | 1 |  |
| Not married | 1.10 | (1.01 to 1.19) | 1.02 | (0.93 to 1.11) |
| **Education** |  |  |  |  |
| None | 1 |  | 1 |  |
| Primary | 1.15 | (0.95 to 1.38) | 1.06 | (0.87 to 1.31) |
| Secondary | 1.35 | (1.14 to 1.61) | 1.12 | (0.93 to 1.35) |
| Tertiary | 1.53 | (1.07 to 2.19) | 1.14 | (0.79 to 1.64) |
| **Time of HIV diagnosis, days** |  |  |  |  |
| Same day | 1 |  | 1 |  |
| Before | 1.16 | (1.08 to 1.26) | 1.25 | (1.15 to 1.35) |
| **CD4 count, cells/mm^3^** |  |  |  |  |
| 0 to 100 | 1.02 | (0.90 to 1.15) | 1.09 | (0.94 to 1.27) |
| 101 to 200 | 1.01 | (0.89 to 1.15) | 0.99 | (0.86 to 1.13) |
| 201 to 350 | 1 |  | 1 |  |
| 351 to 500 | 0.80 | (0.71 to 0.90) | 0.76 | (0.67 to 0.86) |
| ≥501 | 0.71 | (0.63 to 0.80) | 0.66 | (0.58 to 0.75) |
| **WHO clinical stage** |  |  |  |  |
| I/II | 1 |  | 1 |  |
| III | 1.01 | (0.90 to 1.13) | 1.01 | (0.87 to 1.16) |
| IV | 0.92 | (0.68 to 1.26) | 0.94 | (0.68 to 1.31) |
| **BMI, kg/m^2^** |  |  |  |  |
| ≤18.4 | 1 |  | 1 |  |
| 18.5 to <25 | 0.90 | (0.76 to 1.07) | 0.91 | (0.76 to 1.09) |
| ≥25 | 1.05 | (0.88 to 1.25) | 0.94 | (0.77 to 1.16) |
| **Laboratory result^4^** |  |  |  |  |
| Normal | 1 |  | 1 |  |
| Abnormal | 1.15 | (1.02 to 1.31) | 1.11 | (0.97 to 1.27) |
| **Tuberculosis** |  |  |  |  |
| No | 1 |  | 1 |  |
| Yes | 0.97 | (0.82 to 1.15) | 0.88 | (0.68 to 1.15) |
| **Phone availability** |  |  |  |  |
| No | 1 |  | 1 |  |
| Yes | 1.21 | (1.03 to 1.42) | 1.19 | (1.00 to 1.41) |

*aHR, adjusted hazard ratio; ART, antiretroviral therapy; BMI, body mass index; HR, hazard ratio; PHC, primary healthcare level; SHC, secondary healthcare level; SOC, standard of care.*

*^1^Secondary healthcare level comprised ART outpatient departments in one health centre with inpatient capacity in Treat-All and one hospital in SOC.*

*The flexible parametric model (Royston–Parmar models) had five internal knots, and one internal knot for the time-varying covariates health zone and TB.*

All variables tested in univariate analysis were also included in multivariate analysis.
